# Potential of the Liquid Fermentation of Fishery Waste by *Paenibacillus elgii* for Metalloprotease Production

**DOI:** 10.3390/polym14132741

**Published:** 2022-07-05

**Authors:** Chien Thang Doan, Thi Ngoc Tran, Minh Trung Nguyen, Huu Kien Nguyen, Thi Kim Thi Tran, Thi Hanh Nguyen, Thi Phuong Hanh Tran, Van Bon Nguyen, Anh Dzung Nguyen, San-Lang Wang

**Affiliations:** 1 Faculty of Natural Science and Technology, Tay Nguyen University, Buon Ma Thuot 630000, Vietnam; dcthang@ttn.edu.vn (C.T.D.); ttngoc@ttn.edu.vn (T.N.T.); nguyenminhtrung@ttn.edu.vn (M.T.N.); nhkien@ttn.edu.vn (H.K.N.); ttkthi@ttn.edu.vn (T.K.T.T.); ttphanh@ttn.edu.vn (T.P.H.T.); 2 Department of Chemistry, Tamkang University, New Taipei City 25137, Taiwan; 3 Institute of Biotechnology and Environment, Tay Nguyen University, Buon Ma Thuot 630000, Vietnam; nguyenhanh2208.tn@gmail.com (T.H.N.); nvbon@ttn.edu.vn (V.B.N.); nadzung@ttn.edu.vn (A.D.N.); 4 Life Science Development Center, Tamkang University, New Taipei City 25137, Taiwan

**Keywords:** antioxidant, metalloprotease, fishery waste, *Paenibacillus elgii*, protease, tuna heads

## Abstract

This study attempted to use fishery processing wastes to produce protease by *Paenibacillus elgii* TKU051. Of the tested wastes, tuna head powder (THP) was found to be the most effective carbon and nitrogen (C/N) source, and the optimal conditions were as follows: 0.811% THP, 0.052% K_2_HPO_4_, 0.073% MgSO_4_, initial pH of 8.96, incubation temperature of 31.4 °C, and incubation time of 3.092 days to achieve the maximum protease activity of 2.635 ± 0.124 U/mL. A protease with a molecular weight of 29 kDa was purified and biochemically characterized. Liquid chromatography with tandem mass spectrometry analysis revealed an amino acid sequence of STVHYSTR of *P. elgii* TKU051 protease, suggesting that the enzyme may belong to the M4 family of metalloproteases. The optimal activity of the enzyme was achieved at 60 °C and pH 8. *P. elgii* TKU051 protease was strongly inhibited by ethylenediaminetetraacetic acid and 1,10-phenanthroline, indicating its precise metalloprotease property. *P. elgii* TKU051 protease displayed the activity toward casein and raw fishery wastes such as tuna heads, tuna viscera, shrimp heads, and squid pens. Finally, the purified *P. elgii* TKU051 protease could improve the free-radical scavenging activity of fishery wastes. In short, *P. elgii* TKU051 has potential application in eco-friendly approaches to efficiently convert fishery wastes to metalloprotease.

## 1. Introduction

Proteases belong to a class of enzymes that catalyze proteolysis [1,2]. They are by far the most essential category of industrial enzymes, accounting for more than 65% of the commercial enzyme market worldwide [3]. Of enzyme sources, microbial proteases have fascinated the researchers to gain a better understanding of their properties and, eventually, for different applications. Various microbes have been used to produce protease, for example, *Bacillus* [4,5,6], *Paenibacillus* [7,8,9], *Aspergillus* [10,11], *Streptomyces* [12,13], *Pseudomonas* [14,15], and *Brevibacillus* [16,17]. With a range of properties (e.g., thermal stability, alkaliphilic, substrate specificity, and chemical resistance), microbial proteases are used in a wide range of biotech applications such as the leather industry, pharmaceutical industry, detergent industry, food industry, and bioremediation [3,18]. Recently, the use of protease has gotten a lot of interest in producing bioactive protein hydrolysates as a possible alternative to chemical methods that may emit toxic chemicals or residual organic solvents [3,19,20,21]. Given this, proteases have been used as a viable tool for green extraction of the bioactive peptides from fishery wastes to achieve the zero-waste objective [22].

The growth medium comprises 30–40% of the cost of enzyme products [23]. As a result, several researchers have explored strategies to produce microbial proteases utilizing low-cost mediums [24,25,26,27]. Indeed, fishery wastes have been found to hold a lot of promise for this purpose [3]. One of the most significant benefits of using fishery wastes is that they can provide both carbon and nitrogen source; consequently, they can be functionally used as the unique C/N sources [2,28]. Furthermore, the high amount of protein in some fishery wastes (e.g., shrimp head, fish head, fish viscera, squid pen, etc. [22]) may also facilitate the producing protease microbes to consume this kind of nutrient effectively. However, so far, there was no specific medium for the optimal synthesis of proteases from different microbes and different fishery wastes. In general, each microbial strain requires its own set of conditions to generate the maximum amount of fermented products. Therefore, research should be conducted to determine how each strain reacts to different types of fishery wastes to find optimum fermentation conditions for protease production. Of the fishery wastes, tuna heads could be made up for the protease-producing medium, which yields high protease productivity [3,29]. However, not many studies have exhaustively explored the conversion of tuna heads into protease using *Paenibacillus* strains. In the previous study, we observed protease productivity of *P. elgii* TKU051 on the medium containing squid pens as the unique C/N source, indicating the potential of protease production of this strain using the medium containing fishery wastes. Besides, the crude protease cocktail of *P. elgii* TKU051 was also effective in the preparation of bioactive peptides and chitin [2]. However, optimization for the production and the purification of protease from *P. elgii* TKU051 has not yet been performed. Indeed, the production and purification of proteases from *P. elgii* strains are rarely reported [2]. Therefore, this study attempted to optimize protease production from *P. elgii* TKU051 using fishery wastes as the unique C/N source, including tuna heads powder (THP), tuna viscera powder (TVP), white shrimp heads powder (WSHP), tiger shrimp heads powder (TSHP), and squid pens powder (SPP). Further, the *P. elgii* TKU051 protease was also isolated, purified, and characterized. Finally, the protease was used to catalyze THP, TVP, WSHP, TSHP, and SPP to prepare antioxidants.

## 2. Materials and Methods

### 2.1. Materials

*P. elgii* TKU051 was isolated and described in a previous study [2]. Squid pens were bought from Shin-Ma Frozen Food Co. (I-Lan, Taiwan), whereas tuna, tiger shrimp, and white shrimp were bought from Danshui Carrefour supermarket (New Taipei, Taiwan). Subsequently, tuna heads, tuna viscera, and shrimp heads were obtained from the tuna and shrimp. All materials, including squid pens, shrimp heads, tuna heads, and tuna viscera, were dried in the oven (60 °C) and pulverized to powder. Folin’s phenol reagent, tyrosine, 2,2′-azino-bis(3-ethylbenzothiazoline-6-sulfonic acid) (ABTS), 2-mercaptoethanol, Cetrimonium bromide, 2,2-diphenyl-1-picryl-hydrazyl-hydrate (DPPH), trichloroacetic acid (TCA), E-64, phenymethylsulfonyl fluoride (PMSF), ethylenediaminetetraacetic acid (EDTA), 5,5-Dithiobis(2-nitrobenzoic acid) (DTNB), 1,10-phenanthroline were purchased from Sigma-Aldrich (Darmstadt, Germany). All other reagents used in the study were of the highest grade available.

### 2.2. Protease Assay

The assay to determine protease activity was based on a prior study with a slight modification [25]. Briefly, the reaction components consisted of 10 µL enzyme and 90 µL casein (1% in 50 mM Tris-HCl buffer pH 8), and the reaction condition was set at 60 °C for 30 min. Afterward, TCA solution (5%) was added to the mixture to stop the reaction and precipitate the residual casein. Centrifugation (13,000 rpm in 10 min) was used to remove any insoluble particle from the mixture. A mixture of sample solution/Folin’s phenol reagent/sodium carbonate (8:2:15) was transferred into a 96-wells plate and incubated for 20 min in the dark and the color intensity of the solution was determined at 660 nm wavelength. The amount of enzyme required to liberate 1 µmol of tyrosine during one minute proteolytic reaction was described as protease activity.

### 2.3. Screening the Production Conditions

The relevant factors that influence the protease productivity of *P. elgii* TKU051 was explored by the one-factor-at-a-time method in the order of C/N source (THP, TVP, SPP, WSHP, TSHP, casein, and peptone), THP concentration (0.1%, 0.25%, 0.50%, 1%, 1.50%, and 2%), K_2_HPO_4_ concentration (0%, 0.05%, 0.1%, 0.15%, and 0.2%), MgSO_4_ concentration (0%, 0.025%, 0.05%, 0.1%, 0.15%, and 0.2%), pH (5.4, 6.4, 7.4, 8.4, 9.4, and 10.4), temperature (28 °C, 31 °C, 34 °C, 37 °C, 40 °C, and 43 °C), and incubation time (0 day, 1 day, 2 days, 3 days, and 4 days). The original conditions for the experiments were 1% each C/N source, 0.1% KH_2_PO_4_, 0.05% MgSO_4_, pH 7.4, 37 °C, for 3 days. At a time, only one factor was varied while other factors were constant and the condition giving the highest protease activity was chosen for the next experiments.

### 2.4. Optimization of Production

After observing the preliminary result of one-factor-at-a-time experiments, the Box-Behnken design of response surface methodology (RSM) was employed to optimize the response of six independent factors (THP concentration, K_2_HPO_4_ concentration, MgSO_4_ concentration, initial pH, incubation temperature, and incubation time) using R software (Rcmdr package). In a total of 54 runs, each factor was tested at three levels, including low, medium, and high, and were coded as −1, 0, and +1. The result was analyzed by RcmdrPlugin.DoE plugin (R software) to determine the optimum conditions for the protease production process.

### 2.5. Enzyme Purification

The culture supernatant (1.2 L) containing the protease was precipitated with (NH_4_)_2_SO_4_ (60%) and re-dissolved in Tris-HCl buffer (20 mM, pH 8.2). To remove the residual of (NH_4_)SO_4_, the crude enzyme solution was dialyzed against 20 mM pH 8.2 Tris-HCl buffer for 1 day. Then, the crude enzyme was loaded onto a High Q column that was equilibrated with 20 mM pH 8.2 Tris-HCl buffer. The bonded protease was eluted with a gradient of NaCl. The active fraction was collected, dialyzed against 20 mM pH 8.2 Tris-HCl buffer, and then loaded to a DEAE sepharose column. The active fraction was collected by eluting the column with a NaCl gradient; the fraction was then concentrated by the freeze-drying method and loaded onto a Sephacryl S-200 column. Eventually, the purity and biochemical properties of the active fraction were assessed. The molecular weight and purity of the purified protease were determined by SDS-PAGE (sodium dodecyl sulfate-polyacrylamide gel electrophoresis) [19]. The proteolytic activity of purified protease was confirmed on native-PAGE containing 0.05% gelatin. Briefly, the sample was loaded on an acrylamide gel containing 0.05% gelatin, and the electrophoresis was conducted at 140 V and 4 °C. The gel was then incubated in 20 mM pH 8.2 Tris-HCl buffer overnight at 37 °C and stained by Coomassie. After destaining the gel with water, the protease activity band appeared as a clear band.

### 2.6. Biochemical Characterization of Purified Paenibacillus elgii TKU051 Protease

*P. elgii* TKU051 protease activity was measured at 40–90 °C in standard assay conditions, and at pH 3.6–10.6 (citrate buffer (pH 3.6), acetate buffer (pH 4–5), phosphate buffer (pH 6–7), Tris-HCl buffer (pH 8–9), and carbonate buffer (pH 10–10.6)). The experiment on *P. elgii* TKU051 protease substrate specificity was conducted in standard conditions using casein, gelatin, albumin, keratin, hemoglobin, fibrinogen, THP, TVP, TSHP, WSHP, and SPP. The activity toward casein was defined as 100%. The effects of various chemicals on enzyme activity were assessed by adding each chemical to a final concentration of 5 mM, except for laundry detergents and surfactants (final concentration of 1%). The following chemicals were included EDTA, E-64, DTNB, PMSF, 1,10-phenanthroline, 2-mercaptoethanol, ZnCl_2_, FeCl_2_, CaCl_2_, CuCl_2_, MgCl_2_, MnCl_2_, BaCl_2_, cetrimonium bromide, Triton X-100, Tween 40, Tween 20, SDS, Ekos laundry detergent, Amah laundry detergent, and Yeuhyang laundry detergent. The proteolytic activity without added chemicals was defined as 100%.

### 2.7. Hydrolysis of Fishery Wastes

The experiment on fishery wastes hydrolysis of *P. elgii* TKU051 protease was conducted in the standard conditions using THP, TVP, TSHP, WSHP, and SPP as the substrates. The hydrolysis was performed for 0–24 h, and the degree of hydrolysis (DH) of the hydrolysates was calculated based on the amount of the soluble peptide [19].

### 2.8. Assay to Determine Free-Radical Scavenging Activity

Free-radical scavenging activity of hydrolysates was assessed using DPPH and ABTS. Briefly, a 10 µL sample was added to 290 µL DPPH or ABTS solution. Then, the absorbance of the mixture was measured at 517 nm (DPPH) or 750 nm (ABTS). The free-radical scavenging activity was calculated by the following formula:

Free-radical scavenging activity = (A_control_ − A_sample_)/(A_control_ − A_blank_) (%)

where A_control_ is the absorbance of the control, A_sample_ is the absorbance of the sample, and A_blank_ is the absorbance of the blank.

## 3. Results and Discussion

### 3.1. Screening for Optimal Protease-Producing Conditions Using Fishery Wastes as the Unique Carbon and Nitrogen (C/N) Source

**Figure 1:**
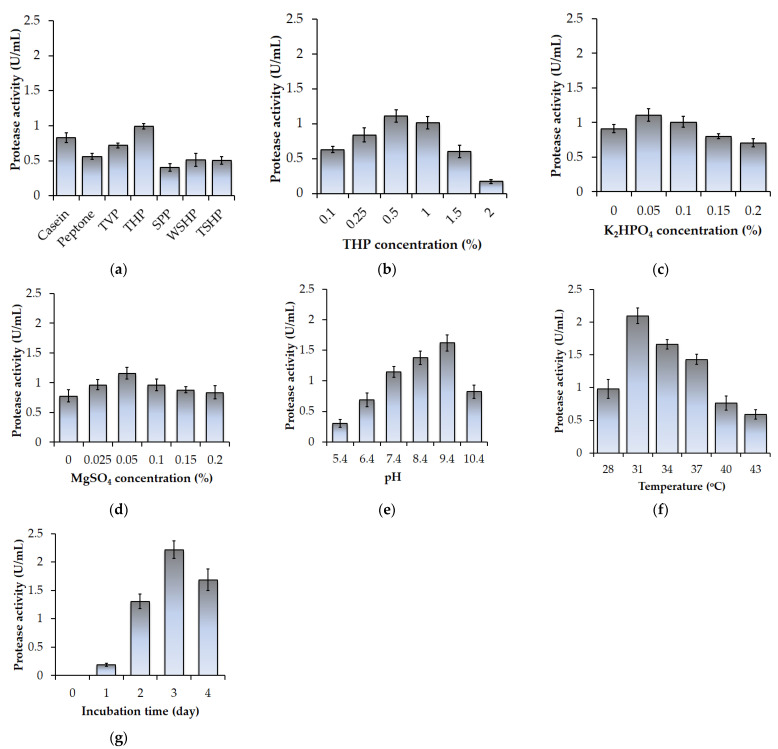
Screening the conditions for protease production using *P. elgii* TKU051. (**a**) the kinds of C/N source; (**b**) THP concentration; (**c**) K_2_HPO_4_ concentration; (**d**) MgSO_4_ concentration; (**e**) initial pH; (**f**) incubation temperature; and (**g**) incubation time. The error bars refer to the standard deviation of three replications.

To explore the influence of the C/N source on protease production, *P. elgii* TKU051 was allowed to grow in various kinds of proteinaceous materials, including TVP, THP, SPP, WSHP, TSHP, casein, and peptone. As shown in Figure 1a, the highest protease activity level was achieved in the culture medium containing THP (0.991 ± 0.042 U/mL), followed by casein (0.829 ± 0066 U/mL), TVP (0.717 ± 0.037 U/mL), peptone (0.561 ± 0.041 U/mL), WSHP (0.512 ± 0.091 U/mL), TSHP (0.506 ± 0.055 U/mL), and SPP (0.405 ± 0.054 U/mL). Fish wastes are rich in specific growth factors and amino acids and have been extensively used as the substrate for microbial protease production [30]. Accordingly, various substances have been indicated as suitable C/N sources for protease production, such as shrimp heads [16], crab shells [19], fish heads [31], and fish viscera [3]. In this study, THP exhibited the highest protease productivity among the tested substrate and thus was selected for the cost-effective production of protease in further experiments. After selecting the best C/N source, various concentrations of THP ranging from 0.1% to 2% were tested. As shown in Figure 1b, the protease productivity progressively increased with an initial increase of THP concentration (from 0.1% to 0.5%), however, a further increase of THP concentration (from 0.5% to 2%) could result in a diminishment of protease productivity. Accordingly, the mediums containing 0.5% and 1% THP showed maximum protease production (1.109 ± 0.088 U/mL and 1.012 ± 0.088 U/mL, respectively, Figure 1b). Since 0.5% THP is cheaper, it was selected for further experiments.

The next experiments were conducted to explore the effect of other parameters on the protease production of *P. elgii* TKU051. There were included K_2_HPO_4_ concentration (0–0.2%), MgSO_4_ concentration (0–0.2%), initial pH (5.4–10.4), incubation temperature (28–43 °C), and incubation time (0–4 day). According to Figure 1c–g, the protease productivity of *P. elgii* TKU051 in different K_2_HPO_4_ concentrations was in the descending order of 0.05% (1.110 U/mL), 0.1% (1.009 U/mL), 0% (0.910 U/mL), 0.15% (0.803 U/mL), and 0.2% (0.705 U/mL); in different MgSO_4_ concentrations was 0.05% (1.162 U/mL), 0.025% (0.968 U/mL), 0.1% (0.964 U/mL), 0.15% (0.880 U/mL), 0.2% (0.837 U/mL), and 0% (0.780 U/mL). The protease productivity of *P. elgii* TKU051 in different initial pHs was pH 9.4 (1.622 U/mL), pH 8.4 (1.377 U/mL), pH 7.4 (1.145 U/mL), pH 10.4 (0.823 U/mL), pH 6.4 (0.693 U/mL), and pH 5.4 (0.302 U/mL); in different incubation temperatures was 31 °C (2.097 U/mL), 34 °C (1.661 U/mL), 37 °C (1.432 U/mL), 28 °C (0.979 U/mL), and 43 °C (0.591 U/mL); in different incubation durations was 3 days (2.217 U/mL), 4 days (1.687 U/mL), 2 days (1.306 U/mL), and 1 day (0.191 U/mL). Taken together, the optimal conditions obtained by one-factor-at-a-time method were 0.5% THP, 0.05% K_2_HPO_4_, 0.05% MgSO_4_, initial pH of 9.4, incubation temperature of 31 °C, and incubation time of 3 days. Accordingly, the highest protease productivity was 2.217 U/mL, which was 2.238-fold higher than that in original conditions using 1% THP as the C/N source. The above results clearly indicate that THP, K_2_HPO_4_, MgSO_4_, pH, temperature, and incubation time had a significant impact on the protease productivity of *P. elgii* TKU051. Therefore, the optimization production of the protease using RSM would be conducted using these factors and conditions.

### 3.2. Optimization Production of Paenibacillus elgii TKU051 Protease

A Box-Behnken experimental design was used to create a quadratic model consisting of 54 runs with six center points. The design matrix and the corresponding results of six independent variables (THP concentration, K_2_HPO_4_ concentration, MgSO_4_ concentration, initial pH, incubation temperature, and incubation time) are shown in Table S1. The influence of the six factors on the protease productivity of *P. elgii* TKU051 was predicted using the following second-order polynomial equation:

Y (U/mL) = 2.3106984 + 0.4152748A + 0.0426658B + 0.0011805C − 0.5694113D + 0.0834766E + 0.1249619F + 0.0303551AB + 0.1373569AC − 0.1831425AD + 0.1016896AE + 0.0594454AF + 0.0713345BC + 0.043509BD − 0.1457045BE − 0.1654353BF − 0.0999189CD + 0.0672872CE − 0.0012648CF + 0.1431749DE − 0.0306081DF − 0.3268234EF − 0.4784022A^2^ − 0.2828647B^2^ − 0.2403675C^2^ − 0.7733526D^2^ − 0.243403E^2^ − 0.671916F^2^

where A, THP concentration (%); B, K_2_HPO_4_ concentration (%); C, MgSO_4_ concentration (%); D, initial pH; E, incubation temperature (°C); F, incubation time (day); Y, predicted protease activity (U/mL).

The regression analysis of the Box-Behnken design is shown in Table 1. The R-squared value of 0.9551 indicates that 95.51% of the total variations can be explained by the model. The adjusted R-squared value of 0.9086 was also relatively high, confirming that the model is significant. The significance of coefficients was determined by the *p*-values; the smaller the value, the more significant it was [32]. In the regression coefficient for linear terms, THP concentration, initial pH, and incubation time were significant for the response. The interaction of THP concentration and initial pH; K_2_HPO_4_ concentration, and incubation temperature; incubation temperature and incubation time contributed to the response at a significant level. Besides, the regression coefficient for quadric terms was highly significant for the response (*p* < 0.01). The F-value of the model was high, indicating that the data was not supported by the null hypothesis. As shown in Table 2, the *p*-values of linear, square, and interaction terms were relatively low (*p* < 0.01), indicating their significance for the response. The F-value (0.8851) and *p*-value (0.624650) of Lack-of-fit also suggest a good fit of the obtained experimental data with the model.

The contour plots and 3D response surface plots of each variable’s pair on protease productivity are given in Figure S1. By using R software (Rcmdr package), the optimal conditions were predicted as follows: 0.811% THP, 0.052% K_2_HPO_4_, 0.073% MgSO_4_, initial pH of 8.96, culture temperature of 31.4 °C, and incubation time of 3.092 days and the predicted maximal activity of *P. elgii* TKU051′s protease was calculated to be around 2.570 U/mL. Under the optimal conditions, *P. elgii* TKU051 exhibited protease productivity of 2.635 ± 0.124 U/mL (Table 3), which is in good agreement with the predicted maximal activity and higher than its protease productivity under unoptimized conditions (2.372-fold) and optimization by one-factor-at-a-time (1.125-fold). In short, this study successfully optimized the protease production process from *P. elgii* TKU051 using tuna heads as the unique C/N source.

### 3.3. Enzyme Purification

**Figure 2:**
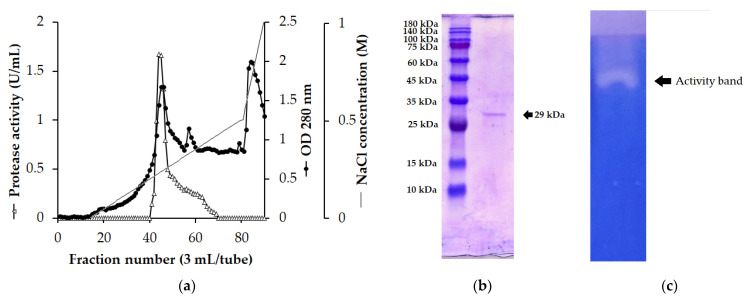
High Q chromatography profile of the crude enzyme (**a**) and SDS-PAGE profile (**b**) and in-gel activity (**c**) of purified *P. elgii* TKU051 protease.

A summary of the *P. elgii* TKU051 protease purification is presented in Table 4. According to the High Q chromatography, only one peak of protease activity was observed at the elution phase (Figure 2a). Consequently, a protease was purified to homogeneity from the culture supernatant with a specific activity of 30.603 U/mg and a purification fold of 46.721-times (Table 4). The molecular weight of *P. elgii* TKU051 protease was estimated to be 29 kDa on SDS-PAGE, and its proteolytic activity was confirmed on native-PAGE containing 0.05% gelatin (Figure 2b,c). The molecular weight of *P. elgii* TKU051 protease was relatively similar to the proteases from *Paeninibacillus* sp. TKU052 (31 kDa) [19], *Paenibacillus* sp. TKU047 (32 kDa) [25], *Paenibacillus* sp. TKU032 (32 kDa) [8], but smaller than proteases from *P. tezpurensis* sp. now. AS-S24-II (43 kDa) [33], *P. larvae* (87 kDa, 74 kDa, 42 kDa, and 40 kDa) [34], *P. polymyxa* EJS-3 (63 kDa) [35], *P. lautus* CHN26 (53.92 kDa) [36], *Paenibacillus* sp. BD3526 (35 kDa) [37], and *Paenibacillus* sp. TKU042 (35 kDa) [7].

The prominent 29 kDa band on SDS-PAGE gel was excised, digested by trypsin, and analyzed by LC-MS/MS. The MASCOT search result (database: Swissprot, Taxonomy: Firmicutes) revealed that a peptide fragment of *P. elgii* TKU051 protease was detected with the amino acid sequences of STVHYSTR (Figure S2), also found in the Bacillolysin protein (a member of the M4 family metalloprotease) from *Brevibacillus brevis* (NPRE_BREBE). This peptide sequence was also compared with known sequences from *Paenibacillus* genus using the BLASTp tool against the non-redundant protein database at NCBI, and it showed a high identity (100%) with M4 family metalloproteases from *Paenibacillus* (WP_069329789) and *P. elgii* (WP_127463081, WP_010498599, and WP_108531393). This suggests that *P. elgii* TKU051 protease may belong to the M4 family metalloprotease (thermolysin-like protease). Likewise, several M4 family proteases produced by *Paenibacillus* strains have been reported [38,39].

### 3.4. Enzyme Biochemical Properties

**Figure 3:**
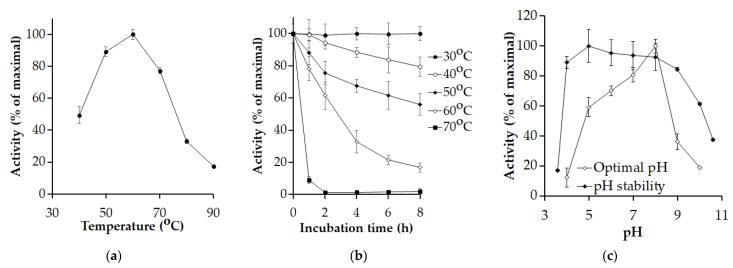
Effect of temperature and pH on the activity of *P. elgii* TKU051 protease. (**a**) optimal temperature; (**b**) thermal stability; and (**c**) optimal pH and pH stability. The error bars refer to the standard deviation of three replications.

*P. elgii* TKU051 protease showed the highest activity at 60 °C (Figure 3a), and its thermal denaturing half-life at this temperature point was 2.83 h (Figure 3b). Similarly, proteases from *Paenibacillus* sp. TKU052, and *P. mucilaginosus* TKU032 have the optimum temperature at 60 °C [8,19]. Meanwhile, Li et al. reported an optimum temperature of 30 °C for a protease from *P. lautus* CHN26 [36]. Because of its excellent performance at 60 °C, *P. elgii* TKU051 protease could be considered as a thermophilic enzyme. After incubating for 8 h, the enzyme can also retain 56%, 79%, and 100% of its initial activity at 50 °C, 40 °C, and 30 °C (respectively). The optimal pH of *P. elgii* TKU051 protease was estimated to be pH 8 in 50 mM Tris-HCl buffer and retained over 80% of its initial activity within a wide pH range of 4–9 (Figure 3c), indicating an advantageous quality of the enzyme for its applications.

The influence of different inhibitors on the activity of *P. elgii* TKU051 protease was determined by pre-treating the enzyme solution with each inhibitor (final concentration of 5 mM) at 20 °C for 1 h. As shown in Table 5, E-64, PMSF, and DTNB exhibited no inhibitory effect, whereas EDTA and 1,10-phenanthroline strongly inhibited the activity of *P. elgii* TKU051 protease (retaining 18% and 5% of its initial activity, respectively). This result indicates that *P. elgii* TKU051 protease is a metalloprotease. Moreover, 1,10-phenanthroline eliminates the proteolytic activity of thermolysin by depriving the enzyme’s Zn^2+^ [40], suggesting that *P. elgii* TKU051 protease is likely a zinc-metalloprotease. This highly agrees with the peptide identification result (described above), which suggests that *P. elgii* TKU051 protease is an M4 family metalloprotease. It is well known that the M4 family is composed mainly of zinc-metalloproteases [41]. So far, most proteases from *Paenibacillus* strains have been found to belong to the metalloprotease group [8,25,35,38], except for a serine protease from *P. tezpurensis* AS-S24-II [33]. Besides, 2-mercaptoethanol exhibited no inhibitory effect, demonstrating that the disruption of disulfide bonds may not affect the activity of *P. elgii* TKU051 protease.

To assess the requirement for metal ions, the influence of various metal ions on *P. elgii* TKU051 protease was explored. Fe^2+^ and Ca^2+^ slightly enhanced the activity of *P. elgii* TKU051 protease (115% and 120%, respectively), whereas Zn^2+^ and Mn^2+^ have a moderate inhibitory effect (85% and 72%, respectively). Cu^2+^ seemed to be an enzyme inhibitor and dramatically inhibited the activity of the enzyme (21%). On removing metal ions in enzyme structure by EDTA and re-incubating the enzyme in the metal ion solutions, *P. elgii* TKU051 protease could recover most of its activity with the appearance of Zn^2+^ (84%) indicating an essential role of Zn^2+^ plays in the enzyme activity. Besides, Ca^2+^, Ba^2+^, and Mn^2+^ could also recover the activity of *P. elgii* TKU051 protease at the lower levels (72%, 53%, and 45%, respectively). Thus, these kinds of metal ions may replace the function of Zn^2+^ to some extent.

Among the tested surfactants, high stability was observed for *P. elgii* TKU051 protease toward Tween 20 (94%) and Tween 40 (95%). *P. elgii* TKU051 protease reduced its activity with the appearance of Triton X-100 (53%) and was inhibited by ionic surfactants such as sodium dodecyl sulfate (SDS) (0%) and cetrimonium bromide (8%). Protease from the M4 family could be incorporated into detergent which effectively eliminated proteinaceous stains from textiles [18], therefore, we herein investigated the compatibility of *P. elgii* TKU051 protease with some commercial laundry detergents. Indeed, *P. elgii* TKU051 protease was compatible with two kinds of detergents (Ekos and Yeuhyang) with the residual activity of 94% and 93% (respectively) whereas, on Amah, the enzyme retained 63% of its initial activity. The inhibitory effect of detergent on metalloprotease activity may be derived from its chelating agent or surfactant components [33]. Based on this result, *P. elgii* TKU051 protease could be a feasible candidate for incorporating with suitable laundry detergents.

The enzyme specificity of *P. elgii* TKU051 protease on protein substrates is listed in Table 6. *P. elgii* TKU051 protease displayed the highest activity towards casein (100%). The activity of *P. elgii* TKU051 protease on albumin (54%), keratin (57%), hemoglobin (47%), and fibrinogen (52%) did not show any significant difference. Besides, *P. elgii* TKU051 protease exhibited the lowest activity towards gelatin (16%). Surprisingly, *P. elgii* TKU051 could also express high proteolytic activity on various raw proteinaceous materials such as TVP (313%), THP (117%), TSHP (166%), WSHP (124%), and SPP (163%) compared to casein substrate. This result indicates that *P. elgii* TKU051 protease may be an excellent candidate to hydrolyze raw fishery materials.

### 3.5. Application of Paenibacillus elgii TKU051 Protease in Hydrolyzing Fishery Wastes and Evaluation of the Antioxidant Activity of Fishery Waste Hydrolysates

**Figure 4:**
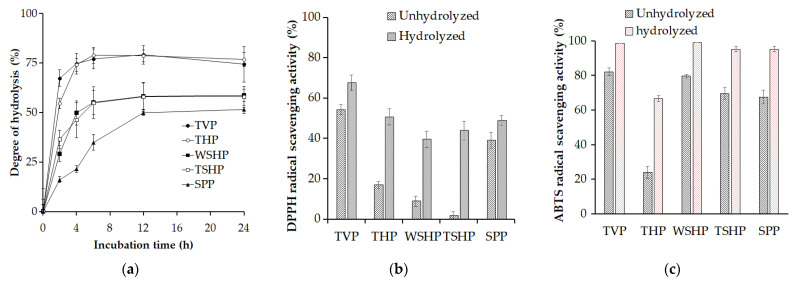
Degree of hydrolysis of fishery wastes treated by *P. elgii* TKU051 protease (**a**) and 2,2-diphenyl-1-picryl-hydrazyl-hydrate (DPPH) radical scavenging activity (**b**) and 2,2′-azino-bis(3-ethylbenzothiazoline-6-sulfonic acid (ABTS) radical scavenging activity (**c**) of fishery wastes hydrolysates. The error bars refer to the standard deviation of three replications.

Fishery waste materials such as TVP, THP, WSHP, TSHP, and SPP were observed to be the most suitable substrates for *P. elgii* TKU051 protease; hence, these materials were used to produce bioactive protein hydrolysates. The hydrolysis was monitored as DH, a measure of the extent of protein hydrolysis. As shown in Figure 4a, the rate of enzymatic hydrolysis reaches the stationary stage around 6–12 h. Besides, the DH values were different for the kinds of materials. According to Figure 4a, the highest DH value was obtained for TVP (79% at 12 h) and THP (79% at 6 h), followed by that for WSHP (58% at 12 h), TSHP (58% at 12 h), and SPP (50% at 12 h). The availability of susceptible bonds and enzyme affinity for diverse substrates may explain the differences in DH values among fishery waste materials [42]. Overall, wastes from tuna (head and viscera) could give a higher DH value than wastes from shrimp (white shrimp heads and tiger shrimp heads) and waste from squid (squid pens).

In living systems, free radicals can cause damage to protein, DNA, and lipid, which can lead to a variety of diseases [43]. As a result, antioxidants could be used to neutralize free radicals. To date, a variety of antioxidant assays have been proposed and used to determine the antioxidant activity. Of antioxidant assays, the DPPH and ABTS assays are two of the most widely used methods for determining antioxidant activity thanks to their convenience [44]. However, it should be noted that these assays could not provide the biological activity of antioxidants. Hence, they are suitable for screening the antioxidant activity of natural extracts [45]. In this study, free-radical scavenging (DPPH and ABTS) activity assays were used to explore the antioxidant activity of the hydrolysates from TVP, THP, WSHP, TSHP, and SPP. As shown in Figure 4b,c, all the hydrolysates revealed higher free-radical scavenging activities than their initial materials in a range of 39–68% against DPPH and 67–99% against ABTS, confirming that *P. elgii* TKU051 protease-mediated hydrolysis on proteins of the tested fishery wastes can liberate the proton-effective peptides that possibly reacted with unstable free-radical (DPPH and ABTS) to convert them into stable forms and terminate the radical chain reaction [19]. Similarly, many investigations found that the protein hydrolysates had higher antioxidant activity than the initial materials [43]. Among hydrolysates, those from TVP exhibited better free-radical scavenging activity than the others, showing 68% of the DPPH radical scavenging activity and 99% of the ABTS radical scavenging activity. Thus, *P. elgii* TKU051 protease could be used as an environmentally friendly and effective tool to extract antioxidants from fishery wastes.

## 4. Conclusions

The optimization production of *P. elgii* TKU051 protease using tuna heads as the unique C/N source resulted in a 2.372-fold increase (2.635 ± 0.124 U/mL) in protease production compared to that in unoptimized conditions. From the THP medium, a metalloprotease with an MW of 29 kDa was isolated, purified, and biochemically characterized. The hydrolysis of fishery wastes such as THP, TVP, WSHP, TSHP, and SPP catalyzed by *P. elgii* TKU051 protease exhibited free-radical (DPPH and ABTS) scavenging activity. Therefore, *P. elgii* TKU051 could be a potential candidate for the production of metalloprotease to convert tuna heads and the preparation of antioxidants.

## Figures and Tables

**Table 1:** Results of regression analysis of Box-Behnken design.

Term	Estimate	Standard Error	t Value	Pr (>|t|)	
(Intercept)	2.3106984	0.0934004	24.7397	<2.2 × 10^−16^	***
A	0.4152748	0.0467002	8.8924	2.303 × 10^−9^	***
B	0.0426658	0.0467002	0.9136	0.3693164	
C	0.0011805	0.0467002	0.0253	0.9800264	
D	−0.5694113	0.0467002	−12.1929	2.941 × 10^−12^	***
E	0.0834766	0.0467002	1.7875	0.0855205	
F	0.1249619	0.0467002	2.6758	0.0127280	*
AB	0.0303551	0.0808871	0.3753	0.7104983	
AC	0.1373569	0.0808871	1.6981	0.1014219	
AD	−0.1831425	0.0571958	−3.2020	0.0035847	**
AE	0.1016896	0.0808871	1.2572	0.2198650	
AF	0.0594454	0.0808871	0.7349	0.4689667	
BC	0.0713345	0.0808871	0.8819	0.3859106	
BD	0.0435090	0.0808871	0.5379	0.5952226	
BE	−0.1457045	0.0571958	−2.5475	0.0171091	*
BF	−0.1654353	0.0808871	−2.0453	0.0510727	
CD	−0.0999189	0.0808871	−1.2353	0.2277696	
CE	0.0672872	0.0808871	0.8319	0.4130605	
CF	−0.0012648	0.0571958	−0.0221	0.9825263	
DE	0.1431749	0.0808871	1.7701	0.0884449	
DF	−0.0306081	0.0808871	−0.3784	0.7082021	
EF	−0.3268234	0.0808871	−4.0405	0.0004206	***
A^2^	−0.4784022	0.0713357	−6.7063	4.084 × 10^−7^	***
B^2^	−0.2828647	0.0713357	−3.9653	0.0005118	***
C^2^	−0.2403675	0.0713357	−3.3695	0.0023591	**
D^2^	−0.7733526	0.0713357	−10.8410	3.844 × 10^−11^	***
E^2^	−0.2434030	0.0713357	−3.4121	0.0021192	**
F^2^	−0.6719160	0.0713357	−9.4191	7.262 × 10^−10^	***

A, THP concentration (%); B, K_2_HPO_4_ concentration (%); C, MgSO_4_ concentration (%); D, initial pH; E, incubation temperature (°C); and F, incubation time (day). Signif. codes: ‘***’ 0.001 ‘**’ 0.01 ‘*’ 0.05; Multiple R-squared: 0.9551; Adjusted R-squared: 0.9086; F-statistic: 20.51 on 27 and 26 DF, *p*-value: 1.292 × 10^−11^.

**Table 2:** Analysis of variance (ANOVA) for the fitted quadratic polynomial model.

	Degrees of Freedom	Sum of Squares	Mean Square	F Value	Pr (>F)
FO(A, B, C, D, E, F)	6	12.5061	2.08435	39.8219	6.794 × 10^−12^
TWI(A, B, C, D, E, F)	15	2.5626	0.17084	3.2639	0.003989
PQ(A, B, C, D, E, F)	6	13.9118	2.31863	44.2979	1.972 × 10^−12^
Residuals	26	1.3609	0.05234		
Lack-of-fit	21	1.0724	0.05107	0.8851	0.624650
Pure error	5	0.2885	0.05770		

A, THP concentration (%); B, K_2_HPO_4_ concentration (%); C, MgSO_4_ concentration (%); D, initial pH; E, incubation temperature (°C); and F, incubation time (day).

**Table 3:** Comparison of *P. elgii* TKU051′s protease production on different culture conditions.

Variable	Before Optimization	After Optimization	
		By One-Factor-at-a-Time	By Response Surface Methodology
THP (%)	1	0.5	0.811
K_2_HPO_4_ (%)	0.1	0.05	0.052
MgSO_4_ (%)	0.05	0.05	0.073
Intitial pH	7.4	9.4	8.96
Temperature (°C)	37	31	31.4
Incubation time (day)	3	3	3.092
Protease activity (U/mL)	1.111 ± 0.041	2.341 ± 0.076	2.635 ± 0.124

**Table 4:** Purification profile of *P. elgii* TKU051 protease.

Purification Step	Total Activity (U)	Total Protein (mg)	Specific Activity (U/mg)	Yield (%)	Purification Fold
Culture supernatant	2153.452	3287.595	0.655	100.000	1.000
(NH_4_)_2_SO_4_ precipitation	995.930	349.447	2.850	46.248	4.351
High Q chromatography	201.220	44.642	4.507	9.344	6.881
DEAE sepharose chromatography	34.489	2.009	17.165	1.602	26.205
Sephacryl S-200 chromatography	24.953	0.815	30.603	1.159	46.721

**Table 5:** Effect of various chemicals on the activity of *P. elgii* TKU051 protease.

Chemical	Relative Activity (%)
None	100 ± 5
EDTA	18 ± 6
E-64	100 ± 4
DTNB	99 ± 9
PMSF	99 ± 5
1,10-phenanthroline	5 ± 7
2-mercaptoethanol	98 ± 7
ZnCl_2_	85 ± 2
FeCl_2_	115 ± 2
CaCl_2_	120 ± 3
CuCl_2_	21 ± 3
MgCl_2_	107 ± 7
MnCl_2_	72 ± 4
BaCl_2_	103 ± 3
^1^ ZnCl_2_	84 ± 3
^1^ FeCl_2_	16 ± 1
^1^ CaCl_2_	72 ± 4
^1^ CuCl_2_	19 ± 7
^1^ MgCl_2_	18 ± 2
^1^ MnCl_2_	53 ± 8
^1^ BaCl_2_	45 ± 4
Cetrimonium bromide	8 ± 1
Triton X-100	53 ± 1
Tween 40	95 ± 2
Tween 20	94 ± 4
SDS	0 ± 0
^2^ Ekos	94 ± 1
^2^ Amah	63 ± 9
^2^ Yeuhyang	93 ± 4

^1^ Enzyme solution was pre-treated by EDTA; ^2^ Commercial laundry detergents.

**Table 6:** Substrate specificity.

Substrate	Relative Activity (%)
Casein	100 ± 6
Gelatin	16 ± 2
Albumin	54 ± 8
Keratin	57 ± 8
Hemoglobin	47 ± 5
Fibrinogen	52 ± 4
TVP	313 ± 23
THP	117 ± 22
TSHP	166 ± 22
WSHP	124 ± 26
SPP	163 ± 24

## Data Availability

Not applicable.

## Supplementary Materials

The following supporting information can be downloaded at: https://www.mdpi.com/article/10.3390/polym14132741/s1, Figure S1. Contour plots (a) and response surface methodology plots (b) for optimization of protease production.; Figure S2. LC-MS/MS spectrum of a peptide fragment originating from *P. elgii* TKU051 protease.; Table S1. Experimental results of the Box-Behnken design.
