# Supplementary material for: Potential of the Liquid Fermentation of Fishery Waste by Paenibacillus elgii for Metalloprotease Production
Source: Polymers (Basel). 2022 Jul 5;14(13):2741. doi: 10.3390/polym14132741 (PMC9268979; doi:10.3390/polym14132741)
Supplement: Supplementary file 1 [file polymers-14-02741-s001.zip › polymers-1743294-supplementary.pdf]

Supplementary Materials

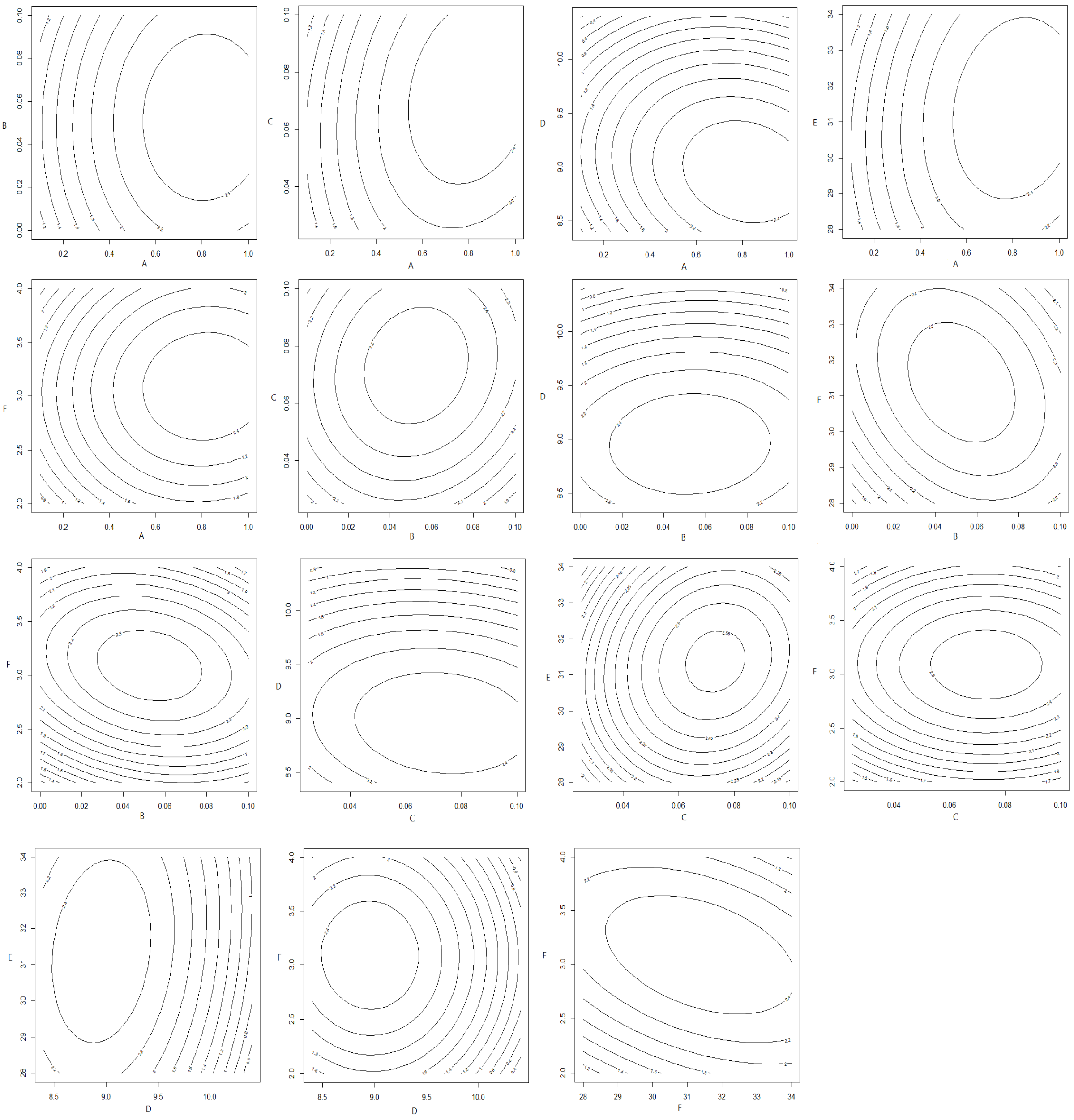

(a)

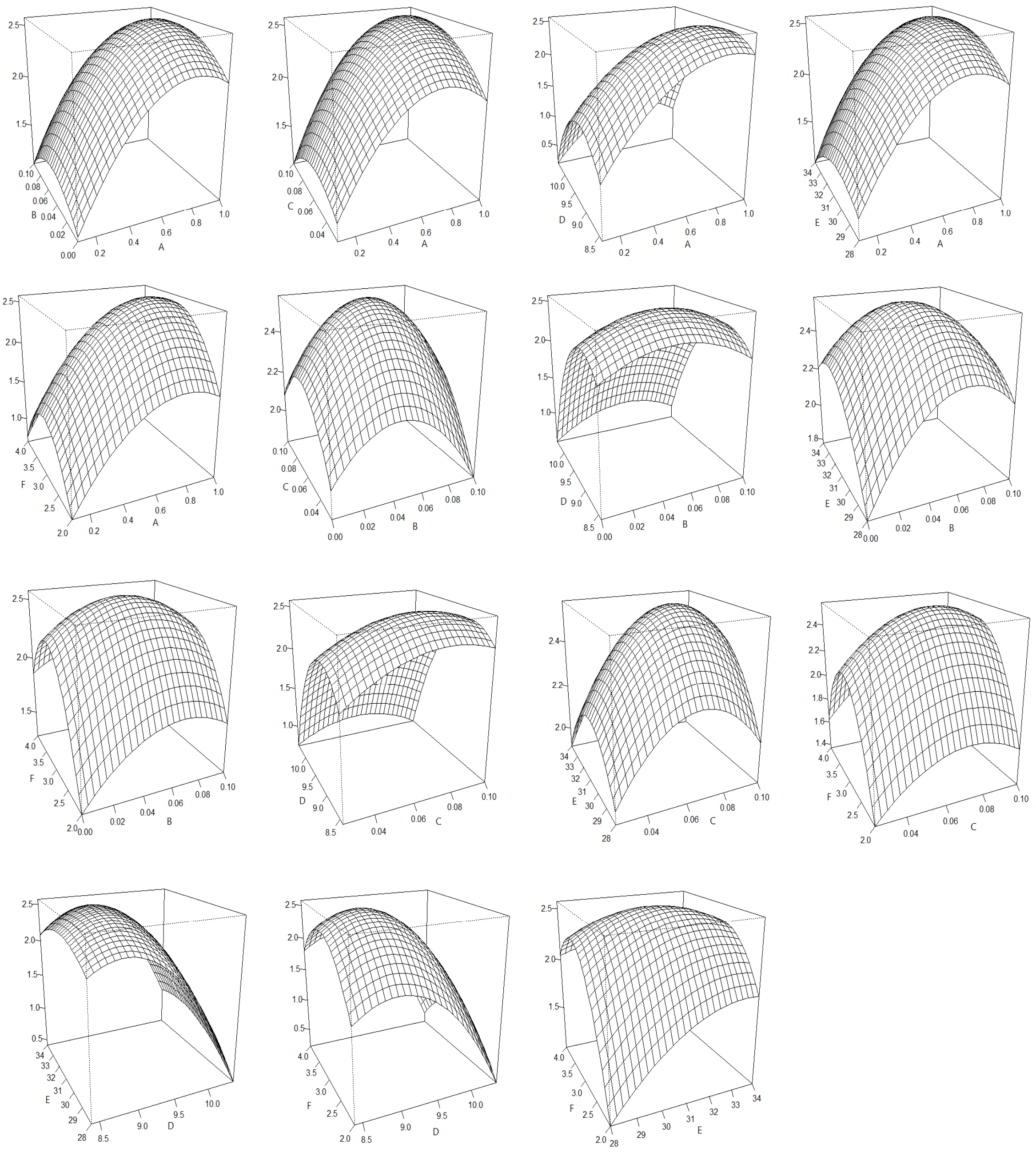

(b)

Figure S1. Contour plots (**a**) and response surface methodology plots (**b**) for optimization of protease production. A, THP concentration (%); B, K_2HPO_4 concentration (%); C, $MgSO_4$ concentration (%); D, initial pH; E, incubation temperature (^{o}C); F, incubation time (day).

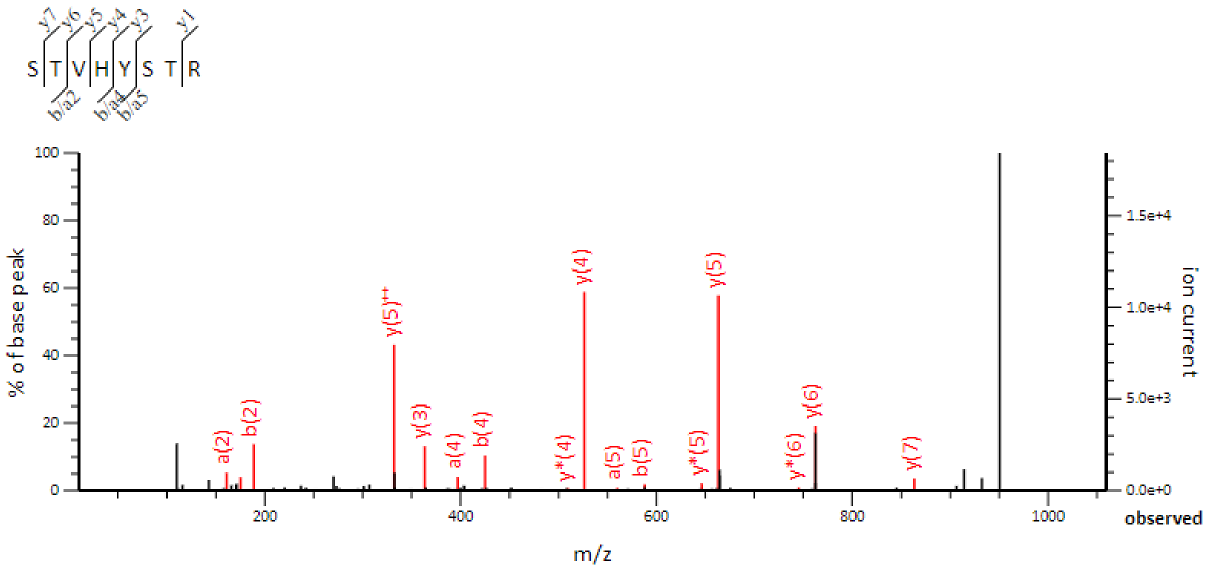

Figure S2. LC-MS/MS spectrum of a peptide fragment originating from *P. elgii* TKU051 protease.

Table S1. Experimental results of the Box-Behnken design.

Run	Uncoded levels						Coded levels						Protease activity (U/mL)
	A	B	C	D	E	F	A	B	C	D	E	F	
1	0.1	0	0.0625	8.4	31	3	-1	-1	0	-1	0	0	0.949
2	1	0	0.0625	8.4	31	3	1	-1	0	-1	0	0	1.892
3	0.1	0.1	0.0625	8.4	31	3	-1	1	0	-1	0	0	0.765
4	1	0.1	0.0625	8.4	31	3	1	1	0	-1	0	0	1.904
5	0.1	0	0.0625	10.4	31	3	-1	-1	0	1	0	0	0.083
6	1	0	0.0625	10.4	31	3	1	-1	0	1	0	0	0.178
7	0.1	0.1	0.0625	10.4	31	3	-1	1	0	1	0	0	0.148
8	1	0.1	0.0625	10.4	31	3	1	1	0	1	0	0	0.289
9	0.55	0	0.025	9.4	28	3	0	-1	-1	0	-1	0	1.534
10	0.55	0.1	0.025	9.4	28	3	0	1	-1	0	-1	0	1.809
11	0.55	0	0.1	9.4	28	3	0	-1	1	0	-1	0	1.020
12	0.55	0.1	0.1	9.4	28	3	0	1	1	0	-1	0	1.720
13	0.55	0	0.025	9.4	34	3	0	-1	-1	0	1	0	1.694
14	0.55	0.1	0.025	9.4	34	3	0	1	-1	0	1	0	1.473
15	0.55	0	0.1	9.4	34	3	0	-1	1	0	1	0	1.589
16	0.55	0.1	0.1	9.4	34	3	0	1	1	0	1	0	1.514
17	0.55	0.05	0.025	8.4	31	2	0	0	-1	-1	0	-1	1.012
18	0.55	0.05	0.1	8.4	31	2	0	0	1	-1	0	-1	1.348
19	0.55	0.05	0.025	10.4	31	2	0	0	-1	1	0	-1	0.018
20	0.55	0.05	0.1	10.4	31	2	0	0	1	1	0	-1	0.091
21	0.55	0.05	0.025	8.4	31	4	0	0	-1	-1	0	1	0.990
22	0.55	0.05	0.1	8.4	31	4	0	0	1	-1	0	1	1.524
23	0.55	0.05	0.025	10.4	31	4	0	0	-1	1	0	1	0.010
24	0.55	0.05	0.1	10.4	31	4	0	0	1	1	0	1	0.008
25	0.1	0.05	0.0625	8.4	28	3	-1	0	0	-1	-1	0	0.876
26	1	0.05	0.0625	8.4	28	3	1	0	0	-1	-1	0	1.929
27	0.1	0.05	0.0625	10.4	28	3	-1	0	0	1	-1	0	0.079
28	1	0.05	0.0625	10.4	28	3	1	0	0	1	-1	0	0.099
29	0.1	0.05	0.0625	8.4	34	3	-1	0	0	-1	1	0	0.771
30	1	0.05	0.0625	8.4	34	3	1	0	0	-1	1	0	1.740
31	0.1	0.05	0.0625	10.4	34	3	-1	0	0	1	1	0	0.057
32	1	0.05	0.0625	10.4	34	3	1	0	0	1	1	0	0.973
33	0.55	0	0.0625	9.4	28	2	0	-1	0	0	-1	-1	0.032
34	0.55	0.1	0.0625	9.4	28	2	0	1	0	0	-1	-1	0.803
35	0.55	0	0.0625	9.4	34	2	0	-1	0	0	1	-1	1.356
36	0.55	0.1	0.0625	9.4	34	2	0	1	0	0	1	-1	1.417
37	0.55	0	0.0625	9.4	28	4	0	-1	0	0	-1	1	1.528
38	0.55	0.1	0.0625	9.4	28	4	0	1	0	0	-1	1	1.457
39	0.55	0	0.0625	9.4	34	4	0	-1	0	0	1	1	1.364

40	0.55	0.1	0.0625	9.4	34	4	0	1	0	0	1	1	0.943
41	0.1	0.05	0.025	9.4	31	2	-1	0	-1	0	0	-1	0.411
42	1	0.05	0.025	9.4	31	2	1	0	-1	0	0	-1	1.141
43	0.1	0.05	0.1	9.4	31	2	-1	0	1	0	0	-1	0.063
44	1	0.05	0.1	9.4	31	2	1	0	1	0	0	-1	1.439
45	0.1	0.05	0.025	9.4	31	4	-1	0	-1	0	0	1	0.593
46	1	0.05	0.025	9.4	31	4	1	0	-1	0	0	1	1.657
47	0.1	0.05	0.1	9.4	31	4	-1	0	1	0	0	1	0.269
48	1	0.05	0.1	9.4	31	4	1	0	1	0	0	1	1.787
49	0.55	0.05	0.0625	9.4	31	3	0	0	0	0	0	0	2.076
50	0.55	0.05	0.0625	9.4	31	3	0	0	0	0	0	0	2.198
51	0.55	0.05	0.0625	9.4	31	3	0	0	0	0	0	0	2.264
52	0.55	0.05	0.0625	9.4	31	3	0	0	0	0	0	0	2.712
53	0.55	0.05	0.0625	9.4	31	3	0	0	0	0	0	0	2.137
54	0.55	0.05	0.0625	9.4	31	3	0	0	0	0	0	0	2.477

A, THP concentration (%); B, K_2HPO_4 concentration (%); C, $MgSO_4$ concentration (%); D, initial pH; E, incubation temperature (°C); and F, incubation time (day).
